# Budget impact analysis and treatment availability with biosimilar TNF inhibitors in rheumatic diseases in Poland: real-world evidence using a nationwide database

**DOI:** 10.1136/ard-2022-223696

**Published:** 2023-06-16

**Authors:** Marcin Stajszczyk, Izabela Obarska, Slawomir Jeka, Bogdan Batko

**Affiliations:** 1 Department of Rheumatology and Autoimmune Diseases, Silesian Center for Rheumatology, Orthopedics and Rehabilitation, Ustroń, Poland; 2 HealthCare System Navigator, Warszawa, Poland; 3 Clinical Department of Rheumatology and Connective Tissue Disease, Collegium Medicum UMK, University Hospital No. 2, Bydgoszcz, Poland; 4 Department of Rheumatology and Immunology, Faculty of Medicine and Health Sciences, Andrzej Frycz Modrzewski University, Kraków, Poland

**Keywords:** Tumor Necrosis Factor Inhibitors, Biosimilar Pharmaceuticals, Adalimumab, Etanercept, Infliximab

## Abstract

**Objectives:**

Although several years have passed since biologic disease modifying antirheumatic drugs were introduced to the market, considerable disparities in access still remain. Tumour necrosis factor inhibitors (TNFi) have proven to be highly effective and safe for treating patients with rheumatic musculoskeletal diseases (RMDs). The emergence of biosimilars is promising for cost reduction and more equitable, widespread access.

**Methods:**

A retrospective budget impact analysis based on final drug prices was conducted using 12 687 treatment courses for infliximab, etanercept and adalimumab. Estimated and real-life savings for public payer were calculated from an 8-year perspective of TNFi use. Data on the treatment cost and on the evolution in the number of patients treated was provided.

**Results:**

From a public payer perspective, the estimated total savings amount to over €243 million for TNFi, with over €166 million attributed to treatment cost reduction in RMDs. Real-life savings were calculated as €133 million and €107 million, respectively. The rheumatology sector generated between 68% and 92% of total savings across models, depending on the adopted scenario. The overall decrease in mean annual cost of treatment ranged between 75% and 89% in the study frame. If all budget savings were spent on reimbursement of additional TNFi, a hypothetical total of almost 45 000 patients with RMDs could be treated in 2021.

**Conclusions:**

This is the first nation-level analysis that shows estimated and real-life direct cost-savings for TNFi biosimilars. Transparent criteria for reinvesting savings should be developed on both a local and an international levels.

**WHAT IS ALREADY KNOWN ON THIS TOPIC**

- Disparities in access to tumour necrosis factor inhibitors (TNFi) are prevalent worldwide.
- On-market introduction of TNFi biosimilars is associated with the potential to favourably impact expenditures and enhance drug access.
- Few long-term data are available that quantify estimated savings based on final drug prices.

**WHAT THIS STUDY ADDS**

- This is a retrospective, nation-level budget impact analysis for TNFi biosimilars based on administrative data extracted from a National Health Fund and final drug prices in an 8-year timeframe.
- We show public payer’s estimated and real-life savings related to the reimbursement of biosimilars and determine the contribution of rheumatology to total savings.
- Access to infliximab, etanercept and adalimumab has not improved remarkably following biosimilar introduction.
- If all budget savings were reinvested into drug reimbursement, up to 20% of biologic eligible patients with rheumatic musculoskeletal diseases could be treated with TNFi in Poland.

**HOW THIS STUDY MIGHT AFFECT RESEARCH, PRACTICE OR POLICY**

- Estimates and real-life savings associated with the biosimilar market presence can vary. This study describes the magnitude of economic and potential healthcare gains at the population level in Poland.
- Our findings may contribute to improved understanding of inequalities in biologics availability in low-income and middle-income countries and may assist decision-making bodies and facilitate data-driven interventions that shape local policy.

## Introduction

Biological disease modifying antirheumatic drugs (bDMARDs), including tumour necrosis factor alpha inhibitors (TNFi), are a well-established treatment modality that has been shown to improve disease-related outcomes in several rheumatic musculoskeletal diseases (RMDs).^1–4^

Although the efficacy and safety of a given DMARD remains crucial in treatment choice, current recommendations also raise the issue of drug cost.^5–7^ In many low-income and middle-income (LAMI) countries (eg, Romania, Poland, Hungary or Bulgaria), high drug prices limit the availability of innovative therapies, including TNFi.^8^ In this setting, biosimilar availability is viewed as a favourable factor that generates savings due to on-market competition and renegotiation of biologic drug prices.

Both disease activity and bDMARD use are associated with socioeconomic factors, drug affordability and reimbursement stringency.^9^ Across Europe, considerable disparities in bDMARD access and eligibility are prevalent.^10^ An analysis conducted in 2014 showed that the annual price of treatment exceeded gross domestic product per capita in 26 countries.^9^ It was also observed that even after adjusting for sociodemographic and clinical characteristics, bDMARD usage remains variable across nations.^11^ Other studies have shown that in low-income nations, a greater rate of patients with moderate-to-high disease activity do not to receive bDMARDs.^12^ These findings are consistent with those of other studies.^9 11 13^

Infliximab (INF), etanercept (ETN) and adalimumab (ADA) biosimilars have been approved for use in indications equivalent to their reference drugs.^1^ Studies have shown that biosimilars may provide substantial financial savings by reducing drug cost expenditures.^14 15^ Compared with the time prior to biosimilar market introduction, the average price (based on list price) per treatment in the entire group of TNFi (both reference drugs and biosimilars) decreased by approximately 13% in the European Union in 2017.^1^ The development of biosimilar TNFi that share the efficacy and safety of the primary agents has led to a more realistic promise of decreased costs and widespread access. However, this optimistic outlook is still limited by several barriers, including doubts over equivalent effectiveness and tolerability and a lack of patient or provider awareness in some healthcare systems or biosimilar naïve countries.^16^ The recent introduction of novel, conveniently administered oral agents is also relevant as a market competitor and therapeutic choice. Updated recommendations regarding Janus kinase inhibitors might re-establish the key role of TNFis in clinical practice.^6^ Compared with individuals in higher welfare nations, patients in Poland face additional barriers to biologic treatment. Restrictive eligibility criteria inconsistent with European Alliance of Associations for Rheumatology (EULAR) recommendations have been partially attenuated within recent years. However, other administrative constraints, such as access to therapy only in centres that sign specific National Health Fund (NHF) contracts, remain relevant. This leads to a limited number of rheumatologists who can treat with biologics, which is compounded by an upper funding limit set by the NHF. Other personal factors, such as travel difficulties, need to be considered. These restrictions persist after the reimbursement of biosimilars, which makes it difficult to increase the number of biologic treated patients. On the other hand, the Polish reimbursement system stimulates competition through local tenders, leading to biosimilar price reductions and reference drug repricing.

This study aimed to assess the impact of the TNFi biosimilars on the public payer budget and examine the availability of TNFis following their market introduction.

## Methods

### Study design and patient population

A retrospective budget impact analysis (BIA) was performed to evaluate the introduction of TNFi biosimilars on the Polish market. INF, ETN and ADA data regarding drug prices, the annual drug budget and the number of patients treated were extracted from the NHF. The total value was calculated for all patients for whom TNFi are reimbursed, including patients with RMDs, rheumatoid arthritis, psoriatic arthritis, ankylosing spondylitis, non-radiographic axial spondylarthritis, juvenile idiopathic arthritis, inflammatory bowel diseases (IBDs), Crohn’s disease, ulcerative colitis and psoriasis. Additionally, the share of rheumatology in the total savings of the public payer was determined. The first biosimilar for INF was available in Poland from 1 January 2014, from 1 July 2016 for ETN and from 1 January 2019 for ADA. This is relevant to the timeframe of drug-specific analyses for which reference costs are calculated at the level of the preceding year. Analyses were performed as recommended by Health Technology Assessment Guidelines, which are consistent with recommendations provided by the International Society for Pharmacoeconomics and Outcomes Research.^17^

The study was based on data calculated annually for a total of 12 687 patients (2438 on INF, 3613 on ETN and 6636 on ADA) throughout the study timeframe. Two models for the assessment of savings were considered—estimated savings, which are a measure of potential savings based on ‘prebiosimilar’ prices and ‘postbiosimilar’ volume—and real-life savings, which show real drug budgets in the prebiosimilar and postbiosimilar era (online supplemental figure 1).

10.1136/ard-2022-223696.supp1 Supplementary data

Data on the annual price of one drug unit and annual cost of treatment for one patient are not based on list prices, which are most commonly used in other relevant studies,^8^ but include the final price of drugs for hospitals resulting from the tender procedures, thereby representing the real cost for the payer. The data cover the entire period of market presence of TNFi biosimilars in Poland.

### Estimated savings of the public payer

Estimated savings associated with INF, ETN and ADA treatment were calculated for actual drug utilisation and for the drug price maintained at the level of the reference drug from the year preceding the coverage of the first biosimilar drug. The presented data cover all clinical indications (table 1) and RMDs separately (table 2). In the alternative scenario of estimated savings, fixed drug cost and constant drug utilisation were both considered at the level of the year preceding access to biosimilar drugs (not shown in detail).

**Table 1:** Estimated savings for the public payer resulting from reimbursement of tumour necrosis factor inhibitor biosimilars in rheumatology, gastroenterology and dermatology for 2013–2021 in Poland

	2013	2014	2015	2016	2017	2018	2019	2020	2021	Total savings
**Infliximab**										
Milligrams of the drug	958 714	1 422 089	1 519 884	1 833 163	2 976 943	4 006 739	4 224 074	3 805 104	4 291 792	
Actual refund value	4.604	4.640	4.337	4.758	7.553	9.261	6.109	3.260	2.827	
Potential refund value	4.604	6.829	7.298	8.803	14.296	19.241	20.284	18.272	20.609	
Estimated savings value	Reference	2189	2.961	4.045	6.743	9.980	14.175	15.012	17.782	**72.887**
**Etanercept**										
Milligrams of the drug	N/A	N/A	4 296 845	4 379 420	4 388 646	4 818 826	5 693 550	5 697 756	6 621 702	
Actual refund value	N/A	N/A	18.455	17.098	13.503	10.401	8.635	7.018	7.094	
Potential refund value	N/A	N/A	18.455	18.810	18.850	20.698	24.455	24.473	28.441	
Estimated savings value	N/A	N/A	Reference	1.712	5.347	10.297	15.820	17.455	21.347	**71.978**
**Adalimumab**	N/A	N/A								
Milligrams of the drug	N/A	N/A	N/A	N/A	N/A	3 304 828	4 003 127	4 000 053	4 818 737	
Actual refund value	N/A	N/A	N/A	N/A	N/A	31.080	11.457	5.951	4.953	
Potential refund value	N/A	N/A	N/A	N/A	N/A	31.080	37.648	37.619	45.318	
Estimated savings value	N/A	N/A	N/A	N/A	N/A	Reference	26.191	31.668	40.365	**98.224**
**Total savings**	**N/A**	**2.189**	**2.961**	**5.757**	**12.090**	**20.277**	**56.186**	**64.135**	**79.494**	**243.089**

Estimated savings are the result of the difference between the potential and actual reimbursement value calculated on the basis of actual drug volume in each year. Potential refund value is based on the prebiosimilar drugs prices. Values are expressed in millions of euro.

N/A, not applicable.

**Table 2:** Estimated savings for the public payer resulting from reimbursement of tumour necrosis factor inhibitor biosimilars in rheumatic musculoskeletal diseases (rheumatoid arthritis, juvenile idiopathic arthritis, psoriatic arthritis, ankylosing spondylitis, non-radiographic axial spondylarthritis) for 2013–2021 in Poland

	2013	2014	2015	2016	2017	2018	2019	2020	2021	Total savings
**Infliximab**										
Milligrams of the drug	164 442	243 922	260 696	352 851	573 008	645 025	682 132	633 945	641 623	
Actual refund value	0.790	0.796	0.744	0.916	1.454	1.491	0.986	0.543	0.423	
Potential refund value	0.790	1.171	1.252	1.694	2.752	3.097	3.276	3.044	3.081	
Estimated savings value	Reference	0.375	0.508	0.778	1.298	1.606	2.290	2.501	2.658	**12.014**
**Etanercept**										
Milligrams of the drug	N/A	N/A	4 481 792	4 377 043	4 286 443	4 367 818	4 377 019	4 807 625	6 535 877	
Actual refund value	N/A	N/A	18.411	17.052	13.467	10.377	8.617	6.969	7.002	
Potential refund value	N/A	N/A	18.411	18.760	18.800	20.649	24.400	24.298	28.072	
Estimated savings value	N/A	N/A	Reference	1.708	5.333	10.272	15.783	17.329	21.070	**71.495**
**Adalimumab**										
Milligrams of the drug	N/A	N/A	N/A	N/A	N/A	2 709 788	3 320 309	3 355 660	4 167 892	
Actual refund value	N/A	N/A	N/A	N/A	N/A	25.484	9.503	4.993	4.284	
Potential refund value	N/A	N/A	N/A	N/A	N/A	25.484	31.226	31.559	39.197	
Estimated savings value	N/A	N/A	N/A	N/A	N/A	Reference	21.723	26.566	34.913	**83.202**
**Total savings**	**N/A**	**0.375**	**0.508**	**2.486**	**6.631**	**11.878**	**39.796**	**46.396**	**58.641**	**166.711**

Estimated savings are the difference between potential and actual reimbursement value calculated on the basis of the actual drug volume in each year. Potential refund value is based on prebiosimilar drug prices. The single unit value is €1 million.

N/A, not applicable.

### Real-life savings of the public payer

Real-life savings are the NHF savings based on the real-life payer’s expenses for the treatment of patients with INF, ETN and ADA after introducing biosimilars. The real-life savings of the public payer for ETN and ADA were calculated for the entire period of biosimilars availability. The real-life savings of the public payer for INF were calculated for 2019–2021 in relation to 2018, in which the expenditure reached its highest level (significantly higher than in the year preceding the reimbursement of the first biosimilar when the numbers of people treated were very small). The presented data cover all clinical indications (table 3) and RMDs separately (table 4). An alternative scenario for INF in a real-life model includes the entire period of biosimilars availability, that is, the reference year for INF 2013 (not shown in detail).

**Table 3:** Real-life public payer savings resulting from the reimbursement of tumour necrosis factor inhibitor biosimilars in rheumatology, gastroenterology, and dermatology for 2013–2021 in Poland

	2013	2014	2015	2016	2017	2018	2019	2020	2021	Total savings
**Infliximab**										
Reimbursement value	4.604	4.640	4.337	4.758	7.553	9.261	6.109	3.260	2.827	
Savings versus reference value	N/A	N/A	N/A	N/A	N/A	Reference	3.152	6.001	6.434	**15.587**
**Etanercept**										
Reimbursement value	19.418	19.204	18.455	17.098	13.503	10.401	8.635	7.018	7.094	
Savings versus reference value	N/A	N/A	Reference	1.357	4.952	8.054	9.820	11.437	11.361	**46.981**
**Adalimumab**										
Reimbursement value	17.946	21.913	26.144	25.081	27.074	31.080	11.457	5.951	4.953	
savings versus reference value	N/A	N/A	N/A	N/A	N/A	Reference	19.623	25.129	26.127	**70.879**
**Total savings**	**N/A**	**N/A**	**N/A**	**1.357**	**4.952**	**8.054**	**32.595**	**42.567**	**43.922**	**133.447**

Savings are calculated as the difference of the reimbursement value in the reference year and that of subsequent years. The single unit value is €1 million.

N/A, not applicable.

**Table 4:** Real-life savings for the public payer due to tumour necrosis factor inhibitor biosimilar reimbursement in rheumatic musculoskeletal diseases (rheumatoid arthritis, juvenile idiopathic arthritis, psoriatic arthritis, ankylosing spondylitis, non-radiographic axial spondylarthritis) for 2013–2021 in Poland

	2013	2014	2015	2016	2017	2018	2019	2020	2021	Total savings
**Infliximab**										
Reimbursement value	0.789	0.796	0.744	0.916	1.454	1.491	0.986	0.543	0.423	
Savings versus reference value	N/A	N/A	N/A	N/A	N/A	Reference	0.505	0.948	1.068	**2.521**
**Etanercept**										
Reimbursement value	19.371	19.157	18.411	17.052	13.467	10.377	8.617	6.969	7.002	
Savings versus reference value	N/A	N/A	Reference	1.359	4.944	8.034	9.794	11.442	11.409	**46.982**
**Adalimumab**										
Reimbursement value	14.733	17.989	21.463	20.529	22.161	25.484	9.503	4.993	4.284	
savings versus reference value	N/A	N/A	N/A	N/A	N/A	Reference	15.981	20.491	21.200	**57.672**
**Total savings**	**N/A**	**N/A**	**N/A**	**1.359**	**4.944**	**8.034**	**26.280**	**32.881**	**33.677**	**107.175**

Savings are calculated as the difference of the reimbursement value in the reference year and that of subsequent years. The single unit value is €1 million.

N/A, not applicable.

### Treatment costs

The average annual costs for each drug and its year-by-year reduction were calculated. To rule out hypothetical inconsistency of data published in the public payer’s reports, sensitivity analyses were performed. The annual costs were determined using two independent analytical methods based on public payer data gathered from different sources (see data source section):

1. Average annual cost of one drug unit (per milligram per year).
2. Average annual cost of treatment for one patient (per patient per year).

Data on treatment costs are presented jointly with the number of biosimilars reimbursed in each year, which reflects the degree of market competition (table 5).

**Table 5:** Infliximab, etanercept and adalimumab prices drop after tumour necrosis factor inhibitor biosimilar entrance based on the average annual cost of one drug unit (mg) and the average annual treatment cost per patient, along with the extent of market competition in 2013–2021 for Poland

	2013	2014	2015	2016	2017	2018	2019	2020	2021
**Infliximab**									
Remicade	Yes	Yes	Yes	Yes	Yes	Yes	Yes	Yes	Yes
Remsima	No	Yes	Yes	Yes	Yes	Yes	Yes	Yes	Yes
Inflectra	No	Yes	Yes	Yes	Yes	Yes	Yes	Yes	Yes
Flixabi	No	No	No	No	No	Yes	Yes	Yes	Yes
Zessly	No	No	No	No	No	No	Yes	Yes	Yes
Cost for 1 mg (€)	4.80	3.26	2.85	2.59	2.54	2.31	1.45	0.86	0.66
% of the reference cost	Reference	68%	59%	54%	53%	48%	30%	18%	14%
Annual cost per pt (€)	7390	5027	4399	3416	4191	3937	2553	1466	1159
% of the reference cost	Reference	68%	59%	46%	56%	53%	34%	20%	14%
**Etanercept**									
Enbrel	Yes	Yes	Yes	Yes	Yes	Yes	Yes	Yes	Yes
Benepali	No	No	No	Yes	Yes	Yes	Yes	Yes	Yes
Erelzi	No	No	No	No	Yes	Yes	Yes	Yes	Yes
Cost for 1 mg (€)	4.32	4.38	4.29	3.90	3.07	2.16	1.52	1.23	1.08
% of the reference cost	N/A	N/A	Reference	91%	72%	50%	35%	29%	25%
Annual cost per pt (€)	7121	7208	7076	6576	5063	3636	2611	2143	1963
% of the reference cost	N/A	N/A	Reference	93%	71%	51%	37%	30%	28%
**Adalimumab**									
Humira	Yes	Yes	Yes	Yes	Yes	Yes	Yes	Yes	Yes
Imraldi	No	No	No	No	No	No	Yes	Yes	Yes
Amgevita	No	No	No	No	No	No	Yes	Yes	Yes
Hyrimoz	No	No	No	No	No	No	Yes	Yes	Yes
Idacio	No	No	No	No	No	No	No	Yes	Yes
Cost for 1 mg (€)	11.87	11.70	11.68	9.79	9.42	9.40	2.86	1.49	1.03
% of the reference cost	N/A	N/A	N/A	N/A	N/A	Reference	30%	16%	11%
Annual cost per pt (€)	7556	7453	7440	6324	6068	6147	2008	1021	746
% of the reference cost	N/A	N/A	N/A	N/A	N/A	Reference	33%	17%	12%

pt, patient.

### Access to treatment

Treatment availability is defined both directly as the number of patients treated and indirectly as a measure of drug exposure expressed as the defined daily dose (DDD) per 1000 inhabitants per year according to WHO.^18 19^ The use of a year instead of a day was due to the unit scale (tables 6 and 7).

**Table 6:** Utilisation of infliximab-containing, etanercept-containing and adalimumab-containing products expressed as DDD per 1000 inhabitants per year, along with the degree of exposure increase in rheumatology, gastroenterology and dermatology for 2013–2021 in Poland

	2013	2014	2015	2016	2017	2018	2019	2020	2021
**Infliximab**									
Milligrams of the drug	958 714	1 422 089	1 519 884	1 833 163	2 976 943	4 006 739	4 224 074	3 805 104	4 291 792
Number of DDD	255 657	379 224	405 302	488 843	793 851	1 068 464	1 126 420	1 014 694	1 144 478
DDD per 1000 inhabitants per year	6.73	9.98	10.66	12.86	20.89	28.12	29.64	26.70	30.12
% of the reference prescription	Reference	148%	158%	191%	310%	418%	440%	396%	448%
**Etanercept**									
Milligrams of the drug	N/A	N/A	4 296 845	4 379 420	4 388 646	4 818 826	5 693 550	5 697 756	6 621 702
Number of DDD	N/A	N/A	613 835	625 631	626 949	688 404	813 364	813 965	945 957
DDD per 1000 inhabitants per year	N/A	N/A	16.15	16.46	16.50	18.12	21.40	21.42	24.89
% of the reference prescription	N/A	N/A	Reference	102%	102%	112%	132%	133%	154%
**Adalimumab**									
Milligrams of the drug	N/A	N/A	N/A	N/A	N/A	3 304 828	4 003 127	4 000 053	4 818 737
Number of DDD	N/A	N/A	N/A	N/A	N/A	1 139 596	1 380 388	1 379 329	1 661 633
DDD per 1000 inhabitants per year	N/A	N/A	N/A	N/A	N/A	29.99	36.33	36.30	43.73
% of the reference prescription	N/A	N/A	N/A	N/A	N/A	Reference	121%	121%	146%

DDD, defined daily dose.

**Table 7:** The potential additional number of patients per year if all real-life savings were spent on reimbursement of additional infliximab, etanercept and adalimumab treatment for 2013–2021 in Poland

	2013	2014	2015	2016	2017	2018	2019	2020	2021
**Infliximab**									
Real number of pts (all)	623	923	986	1393	1802	2352	2393	2224	2438
Potential additional number of pts (all)	N/A	N/A	N/A	N/A	N/A	N/A	1234	4092	5549
Total potential number of pts (all)	623	923	986	1393	1802	2352	3627	6316	7987
Real number of pts (REUMA)	106	157	168	313	365	419	398	332	322
Potential additional number of pts (REUMA)	N/A	N/A	N/A	N/A	N/A	N/A	198	646	921
Total potential number of pts (REUMA)	106	157	168	313	365	419	596	978	1243
**Etanercept**									
Real number of pts (all)	2727	2664	2608	2600	2655	2851	3292	3250	3553
Potential additional number of pts (all)	N/A	N/A	N/A	206	978	2214	3760	5337	5787
Total potential number of pts (all)	2727	2664	2608	2806	3633	5065	7052	8587	9340
Real number of pts (REUMA)	2717	2654	2598	2587	2655	2851	3292	3250	3553
Potential additional number of pts (REUMA)	N/A	N/A	N/A	207	976	2209	3751	5337	5787
Total potential number of pts (REUMA)	2717	2654	2598	2794	3631	5060	7043	8587	9340
**Adalimumab**									
Real number of pts (all)	2375	2940	3514	3966	4462	5056	5706	5837	6636
Potential additional number of pts (all)	N/A	N/A	N/A	N/A	N/A	N/A	9772	24 606	35 009
Total potential number of pts (all)	2375	2940	3514	3966	4462	5056	15 478	30 441	41 645
Real number of pts (REUMA)	1914	2369	2832	3124	3570	4097	4699	4937	5673
Potential additional number of pts (REUMA)	N/A	N/A	N/A	N/A	N/A	N/A	7959	20 066	28 407
Total potential number of pts (REUMA)	1914	2369	2832	3124	3570	4097	12 658	25 003	34 080

These data include the number of patients treated in all clinical indications (all) and separately the number of patients with rheumatic musculoskeletal diseases (rheumatoid arthritis, juvenile idiopathic arthritis, psoriatic arthritis, ankylosing spondylitis, non-radiographic axial spondylarthritis) (REUMA). The potential additional number of patients has been calculated based on real-life savings in each year and the average annual treatment cost per patient in the corresponding year.

N/A, not applicable; pts, patients.

**Figure 1:**
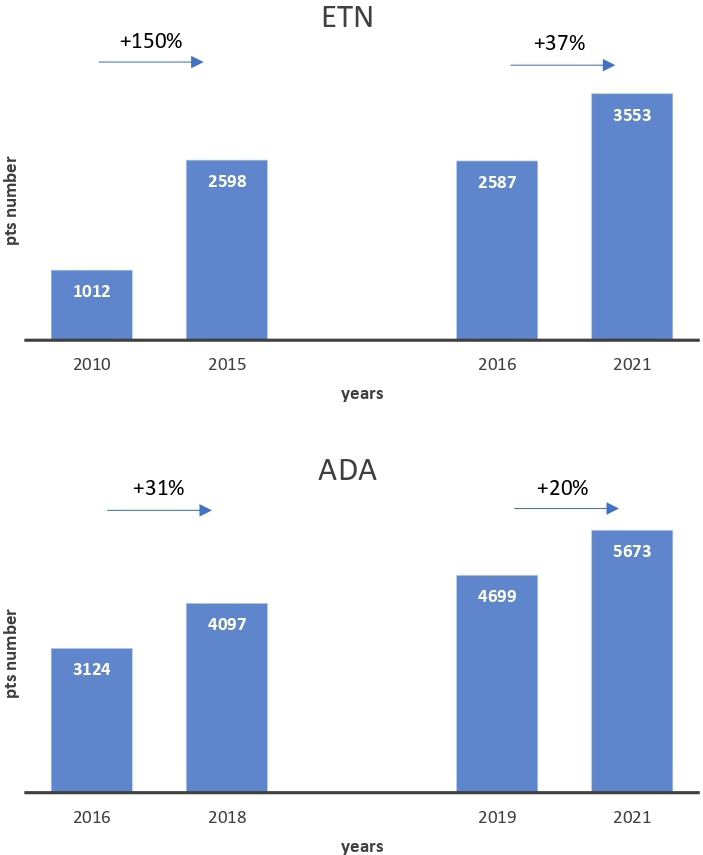
The rise of etanercept (ETN) and adalimumab (ADA) use in patients with rheumatic musculoskeletal diseases (rheumatoid arthritis, juvenile idiopathic arthritis, psoriatic arthritis, ankylosing spondylitis, non-radiographic axial spondylarthritis) in Poland during the biosimilar competition era (ETN, 2016–2021; ADA, 2019–2021) and in the corresponding period of market exclusivity of reference drugs (ETN, 2010–2015; ADA, 2016–2018).

Based on the number of patients treated, ETN and ADA growth in patients with RMDs during the biosimilars competition era (ETN, 2016–2021; ADA, 2019–2021) was compared with growth in the corresponding period of market exclusivity of reference drugs (ETN, 2010–2015; ADA, 2016–2018) (figure 1). Data for INF would not be reliable due to the very small numbers of patients treated prior to 2014 and patients with RMDs in general.

**Figure 2:**
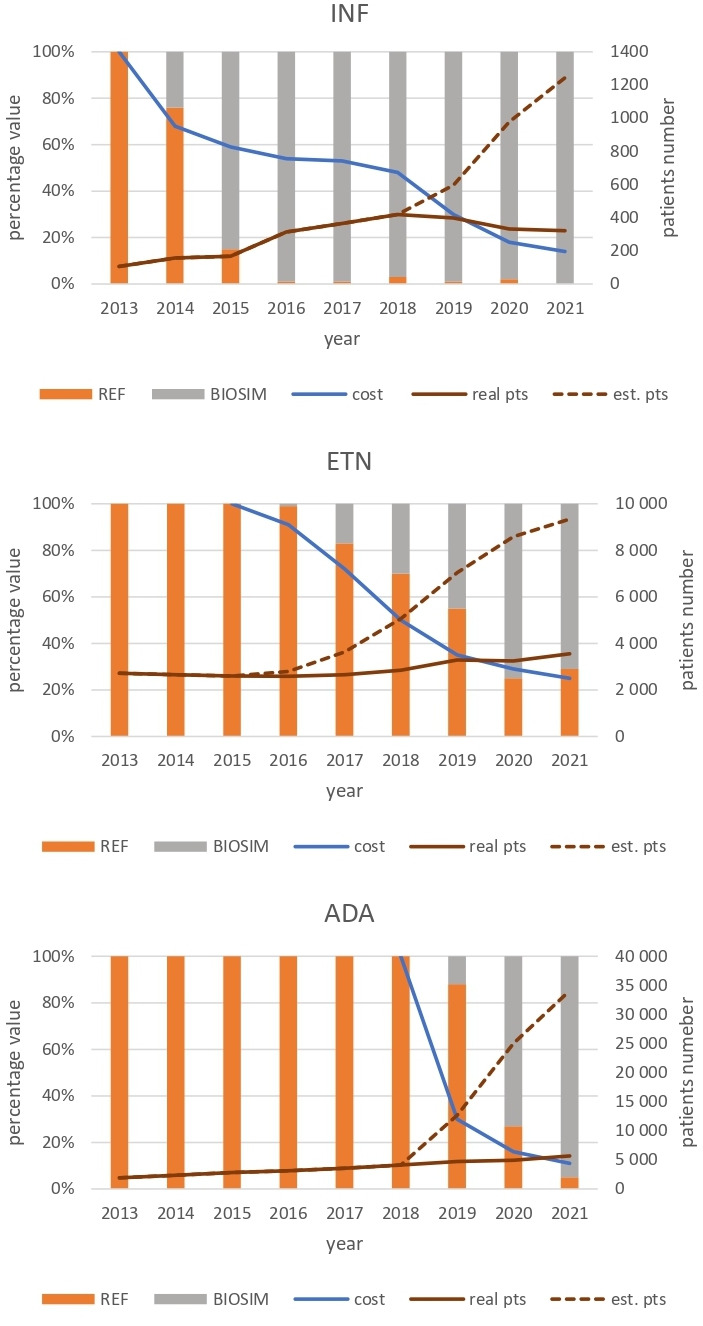
Line graph illustrating the number of patients treated (real pts; *solid line, brown*) and potential total number of patients treated annually (est. pts; *dotted line, brown*) if all real-life budget savings were spent on reimbursement of additional infliximab (INF), etanercept (ETN) and adalimumab (ADA) in RMDs (rheumatoid arthritis, juvenile idiopathic arthritis, psoriatic arthritis, ankylosing spondylitis, non-radiographic axial spondylarthritis) for 2019–2021 in Poland. The market share of the reference biologic (REF) and biosimilars (BIOSIM) is based on reimbursement value data (stacked clustered chart). Additionally, the percentage treatment cost reduction due to the reimbursement of biosimilar medicines (cost; *solid line, blue*) is shown.

The potential additional total number of patients treated per year if all real-life savings in 2019–2021 were spent on reimbursement of additional INF, ETN and ADA is shown in table 7. This was calculated based on real-life savings in each year and the average annual treatment cost per patient in the corresponding year. The market share of the reference biologic compared with biosimilars was also calculated using reimbursement data (figure 2).

### Data source

Data on the annual public payer expenses related to reimbursement of INF, ETN and ADA, the number of patients treated and the number of drug units were sourced from different published NHF data sets: the NHF Council resolutions, the Department of Drug Management announcements and the NHF Statistics Portal.^20–22^

Population size estimates were based on the average for the analysed period. Currency conversion was based on average euro (€) exchange rates in the last 5 years (1 Polish Zloty=€4.4124).

## Results

### Estimated savings of the public payer

Total savings were estimated at €243.083 million (table 1), of which €166.711 million were generated within the rheumatology sector (table 2). In the case of an alternative scenario that assumed no increase in the number of patients treated during the biosimilar reimbursement period, total and RMDs savings were reduced by 54% and 38%, respectively (total estimate of €111.683 million, €103.816 million for RMDs). The treatment of rheumatic diseases accounts for over 68% of total savings in the basic scenario and up to 92% in the alternative scenario.

### Real-life savings of the public payer

The total real-life public payer savings were calculated as €133.447 million (details provided in table 3), of which €107.175 million were generated by rheumatic patients (see table 4).

The alternative scenario, which uses as reference value INF budget at the year prior to biosimilar coverage instead of 2018, yielded considerably different results. A net positive for INF was observed only for 2015, 2020 and 2021, while in the remaining years, an increase in expenses related to the number of patients treated was observed. Consequently, instead of savings for INF, we obtain an increase in the payer’s expenses of €5.915 million. The total savings in this scenario are then reduced to €111.948 million. Similarly, in the RMD population, the NHF recorded an increase in expenditures related to INF reimbursement of €1.036 million, and the total savings decreased to €103.619 million. The rheumatology sector accounts for over 80% and 92% of savings, depending on the scenario.

### Treatment costs

The overall decrease in the mean annual cost of treatment, calculated using the drug unit cost, was estimated at 86%, 75% and 89% for INF, ETN and ADA, respectively, within the entire study timeframe (table 5). Price reduction calculated using average annual treatment cost for one patient was similar (86%, 72% and 88%, respectively), thus not indicating relevant hypothetical inaccuracies in the data reported by the NHF. The greatest market competition concerns drugs containing INF and ADA (five medicinal products) and is lower in the case of ETN (three medicinal products). In 2021, in absolute terms, the lowest annual cost of therapy applies to ADA, which represents approximately 64% and 38% of the cost of INF and ETN treatment, respectively.

### Access to treatment

Overall, access to TNFi treatment slightly increased after the introduction of biosimilars and was most pronounced for INF, and these observations were consistent across both outcome measures: DDD per 1000 inhabitants per year and number of patients (for details, see tables 6 and 7).

In the case of RMDs, compared with the number of patients treated in the last year of market exclusivity, we observe an increase in INF (8 years on the market) by approximately 200% (absolute value+216 pts) and approximately 36% (+955 pts) and 38% (+1 576 pts) in the case of ETN (6 years on the market) and ADA (3 years on the market), respectively. However, when comparing the growth of the number of ETN-treated and ADA-treated patients during the biosimilar reimbursement period to the corresponding period of market exclusivity of the reference drug, we find that the observed percentage change is negative. Moreover, the absolute growth is lower for ETN (−552 pts) and almost equal for ADA (+1 pt) (figure 1).

If all budget real-life savings were spent on reimbursement of additional INF, ETN and ADA treatments, approximately 1234–5549, 3760–5787 and 9772–35 009 hypothetical more patients could be treated in Poland every year in 2019–2021, respectively. This is about 1.5–3.3-fold, 2.1–2.6-fold and 2.7–6.3-fold more total patients per year by drug in the indicated years in all indications. Given the contribution of rheumatology to the total NHF savings, the hypothetical increase in the number of patients with RMDs treated with INF, ETN and ADA is equal to 198–921, 3751–5787 and 7959–28 407 in 2019–2021 per year, respectively (table 7).

The market share analysis indicates a yearly increase and ultimately dominant market share for biosimilars. In 2021, the use of reference drugs accounted for close to 0% for INF, 5% for ADA and 29% for ETN. The number of patients with rheumatic diseases treated with TNFi biosimilars could be much greater if we consider the decrease in drugs prices and the savings achieved (figure 2).

## Discussion

This is the first complex, retrospective, nation-level BIA covering all available TNFi biosimilars that provide an exact analysis of public payer savings associated with their market introduction. We provide detailed data on both estimated and real savings generated by biosimilar presence on the market in an 8-year timeframe from a public payer perspective. By reporting both the number of patients treated and drug utilisation data, potential extrapolation of projected and real-life savings can be performed despite cross-country differences. It is important to emphasise that on a healthcare system level, the majority of savings are generated by TNFi biosimilar use in rheumatic diseases, which reflects the predominance of patients with RMDs among biosimilar users.

In Poland, biologic reimbursement is based on a set of eligibility criteria published by the Ministry of Health. Fulfilment of all conditions facilitates patient enrolment in a dedicated drug programme (DP), which enables hospitals to have their costs covered by NHF. DPs are dedicated to several RMDs, and various drugs with different mechanisms of action can be reimbursed under one DP. Tendering related to the purchase of biologic drugs takes place at the hospital level and is neither regional nor central. If more than one medical product with the same active substance is reimbursed, the active substance product is mandated to undergo a tender procedure. Switching to the drug selected in a tender is mandatory but not automatic at the pharmacy level (eg, reference to biosimilar, biosimilar to reference, biosimilar to biosimilar). Each hospital has the right to provide patients with a specific product if a return to the original is requested due to suspicion of intolerance or lower effectiveness. Physicians are obliged to inform patients if the drug is switched and provide additional information regarding equivalence with the reference drug. All patients are also informed about the possibility of undergoing a reverse switch (in case of related concerns). Therefore, to be used in practice, the reference drug must be selected in a tender, which also stimulates price reduction.

We observed a high degree of integration for biosimilars in the treatment of rheumatic diseases in Poland, which, as described above, is based on observations regarding new prescriptions and non-medical switching from a reference product. In part, this reflects mutual agreement on a strategy supported by the public payer and experts from Polish Society for Rheumatology (PTR) and effective transition and acceptance by the specialist provider. The PTR has promoted educational programmes for rheumatologists and patients in line with EULAR recommendations^23^ and maintained a generally favourable image of biosimilars in media communications. As part of the biosimilar adoption programme, the PTR and NHF have cooperated on the final shape of incentives to use INF, ETN and ADA (biosimilar or original) over other innovative therapies, which includes other TNFi. If the expected benefit and safety of different therapeutic options is comparable, treating patients with the afore-mentioned drugs provides hospitals with higher remuneration for healthcare services. A fixed upper limit for the final drug prices of INF, ETN and ADA (based on nationwide average prices) is an additional condition that guarantees benefits related to the provision of services. It also stimulates the repricing of reference drugs.

Together, these efforts culminate in significant savings. In rheumatology alone, it can be estimated that potential savings of the public payer range from €104 to €167 million, depending on the adopted scenario. These projections include different drug consumption levels but savings estimations assuming actual drug utilisation (ie, year-on-year growth with a trend not exceeding that noted prior to biosimilar reimbursement) credibly reflects the payer’s savings.

Estimated savings are derived from the number of treated patients and drug cost reduction. However, the calculation of estimated savings does not mean that the payer has obtained a net benefit (ie, lower annual expenses related to reimbursement of a given drug compared with the prior year). The increase in the number of treated patients may be high enough that the payer’s expenses may remain at a similar level as that prior to the price drop. Ideally, this is the most desirable scenario as it reflects a full reinvestment of savings for the treatment of a greater number of patients. The payer’s real savings appear when the decrease in treatment costs is not used to treat a correspondingly larger number of patients. By financing treatment at an equivalent accessibility level (ie, as prior to the biosimilar era), the public payer achieves significant savings, but this strategy does not facilitate potential improvement in disease control and associated socioeconomic burden on a population level. Indirect costs associated with the progression of disease-related disability can constitute the majority of expenses in rheumatic disease.^24^ Enabling access to biosimilars earlier in the disease course and to an increased number of patients is likely to improve the state of RMD control population-wise, especially since biologic availability in Poland is low. Our analysis provides evidence that savings generated due to the price drop of TNFi were mainly used by the public payer to reduce expenses by over €100 million among patients with RMDs rather than to increase drug access.

The current analysis is difficult to compare with other reports. Few data are available regarding biosimilar market presence and budget impacts.^25^ Prior projections are based on fixed degrees of market penetration and expected price reductions estimated at a time point when biosimilars were being introduced to the market, often covered only a single drug or clinical indication.^15 26–29^ We identified only one study that included a savings analysis based on real drug prices over the long term.^30^ Although conducted on a national level, the study (corresponding to our estimated savings model) has several limitations, including no reporting of patients treated or drug unit utilisation and no calculation of real-life savings, which precludes a direct comparison. In the case of Poland, the presented data indicate that estimated savings were achieved primarily due to the reduced cost of therapy. For other countries, a smaller reduction in the price of biologic drugs can yield economic gains given a larger sample of treated patients.

We observed that following their market introduction, INF and ADA biosimilars practically replaced their reference drugs in the third year of availability. Only the market share for the reference ETN remained significant due to successful competition in hospital-level tenders. Access to biosimilars has also resulted in downward repricing of reference drugs in Poland. This applies with the greatest extent to ETN, and less to INF and ADA (data not presented in detail). However, this scenario is not replicated on a similar level in other countries and likely depends on a variety of factors (eg, local economic policy, healthcare system construction). Notably, despite having the shortest presence on the market, the greatest savings are generated by ADA, which is likely the least expensive TNFi treatment. The market penetration of biosimilars in Poland reflects a similar trend to that observed in the Nordic countries, along with the effective market competition of the reference ETN in Norway, winning the central tender in 2020.^31^

From a healthcare perspective, savings generation requires a high amount of biologic users. We observed that the uptake of biosimilars within the Polish market is very high. In contrast, market shares for INF were only 2% after 5 years in the UK, Germany, France, Spain and Italy.^32^ The Polish actual scenario is most similar to Norway, although with no central tendering.^33^ We noted the greatest increase in drug availability for INF, with patients’ access to treatment improving over four-fold between 2014 and 2018. INF market penetration mainly covers patients with IBD (almost 90% of the INF market in Poland), as the setting of INF use in RMDs is quite specific. According to the current reimbursement criteria, a patient may be treated with up to two (ineffectiveness) or three (intolerance) TNFis; hence, physicians tend to favour less immunogenic drugs, which results in an expectedly low penetration of INF in patients with RMDs.

Less pronounced figures are noted for the availability of ETN and ADA. For both drugs, the increase in the number of patients does not exceed 40% and should be interpreted in context of a small set of users prior to the biosimilar era. Based on data published by Jensen *et al*,^34^ we estimated that the increase in ADA consumption in Denmark (12 months prior to and after biosimilar reimbursement) was close to 30%, as compared with 21% in the corresponding period for Poland. In line with our findings, the authors showed considerable real-life savings of about 80% when compared with the prior (annual) payer budget. Similarly, access to treatment in Norway has considerably improved for ADA (>200% for 2018–2021) and ETN (~50% for 2016–2021).^31^ When comparing the Polish scenario with both studies,^31 34^ the rate of improvement in biologic accessibility is partially comparable. However, the absolute number of initial therapy users leads to different implication on both an economic and patient level. To illustrate this, we quantified the number of 40 mg ADA pens (from the Jensen study) into DDD per 1000 inhabitants (per country, per year). We observed that the initial drug utilisation was close to 28 times greater than in Poland. This is a remarkable disparity, which impacts savings calculation that could infer for, for example, treatment-related improvements in work ability of the RMD population. It also illustrates the real-life setting of LAMI countries.

If all budget savings were spent on reimbursement of additional TNFis, a total of over 46 000 additional patients could benefit from treatment. For the rheumatology sector, this represents over 35 000 additional biologic users, giving a total of almost 45 000 biologic patients treated annually. Adopting two conservative thresholds of 40% and 60% for the rate of biologic eligible patients with RMDs—defined as not achieving at least low disease activity under conventional treatment^35 36^ and based on epidemiological data^35 37 38^ with a total population of patients with inflammatory arthritis in Poland estimated at approximately 550 000—yields an estimate of 14%–20% of eligible patients that might be treated with TNFi instead of the current 3%–4%. This is predicated on full reinvestment of savings into biologic reimbursement. Although biologic eligibility does not equate to TNFi eligibility, if we assume a conservative rate of 20%, the estimated number of treated patients could be achieved in practice.

The current biologic reimbursement regulations in Poland do not enable enhanced drug access. The observed increase in the number of treated patients is still mainly derived from very low rates of initial drug availability. For the most cost-effective subcutaneous therapies (eg, ETN or ADA) to become widely available, extension of prescription mandate to all retail pharmacies and access for all rheumatologists (rather than limitation to selective inpatient care) appears necessary. Transparent criteria for reinvesting savings due to benefit-sharing programmes should be developed; otherwise, patients who have been promised accessible and affordable therapy will suffer.^39^ A study by Jensen *et al* from Denmark has shown that cost reduction is still achievable, even when maximising treatment access is prioritised.^34^ On a population-level, developing similar policies may facilitate the expected effects of biosimilar market presence for the Polish setting, with improvement of healthcare outcomes,^40^ as has been reported in other countries.^41^

### Strengths

The major advantage of this analysis is an exact calculation based on the public payer’s data, which includes final (not list) drug prices derived from all treatments on the population level. Since the vast majority of healthcare in Poland is financed by NHF, such sourcing of data represents a reliable approach to describe the nation-level effect of biosimilar market introduction. Determination of the rheumatology sector contribution to the total results is an added advantage.

### Limitations

The current analysis may be limited by the hypothetical inconsistency of data published by the public payer. To evaluate the discrepancies across different NHF records, treatment costs and availability were calculated in two different approaches using various NHF sources. We did not observe relevant differences in the obtained calculations. It should be noted that the share of rheumatology in total savings for individual countries will vary depending on the number of patients treated in each clinical indication.

## Conclusions

This study demonstrates that biosimilar-related reduction in TNFi prices mainly led to reduced expenditure within the RMDs sector of Polish healthcare. No relevant increase in treatment availability was observed due to limited savings reinvestment into additional TNFi treatments. If no systemic changes towards a patient-centric healthcare model are undertaken, we can expect only a modest yearly increase in biologic drug access, for which the observed rate of change is still likely driven by low starting drug availability. Our findings illustrate the extent of real-life (in contrast to the expected) patient-level benefits in an LAMI country, and demonstrate how a reductive fiscal policy (with stringent reimbursement and restrictive prescription) constricts biologic accessibility.

## Data Availability

Data are available in a public, open-access repository. The source raw data are available through published NHF data sets: the NHF Council resolutions, the Department of Drug Management announcements and NHF Statistics Portal.

## References

[1] Smolen JS , Goncalves J , Quinn M , et al . Era of Biosimilars in rheumatology: reshaping the Healthcare environment. RMD Open 2019;5:e000900. 10.1136/rmdopen-2019-000900 31245050 PMC6560670

[2] Aaltonen KJ , Virkki LM , Malmivaara A , et al . Systematic review and meta-analysis of the efficacy and safety of existing TNF blocking agents in treatment of rheumatoid arthritis. PLoS One 2012;7:e30275. 10.1371/journal.pone.0030275 22272322 PMC3260264

[3] Fleischmann R , Tongbram V , van Vollenhoven R , et al . Systematic review and network meta-analysis of the efficacy and safety of tumour necrosis factor inhibitor–methotrexate combination therapy versus triple therapy in rheumatoid arthritis. RMD Open 2017;3:e000371. 10.1136/rmdopen-2016-000371 28123782 PMC5237767

[4] Callhoff J , Sieper J , Weiß A , et al . Efficacy of TNFα blockers in patients with Ankylosing Spondylitis and non-radiographic axial Spondyloarthritis: a meta-analysis. Ann Rheum Dis 2015;74:1241–8. 10.1136/annrheumdis-2014-205322 24718959

[5] Gossec L , Baraliakos X , Kerschbaumer A , et al . EULAR recommendations for the management of Psoriatic arthritis with pharmacological therapies: 2019 update. Ann Rheum Dis 2020;79:700. 10.1136/annrheumdis-2020-217159 32434812 PMC7286048

[6] Smolen JS , Landewé RBM , Bergstra SA , et al . EULAR recommendations for the management of rheumatoid arthritis with synthetic and biological disease-modifying Antirheumatic drugs: 2022 update. Ann Rheum Dis 2023;82:3–18. 10.1136/ard-2022-223356 36357155

[7] Ramiro S , Nikiphorou E , Sepriano A , et al . ASAS-EULAR recommendations for the management of axial Spondyloarthritis: 2022 update. Ann Rheum Dis 2023;82:19–34. 10.1136/ard-2022-223296 36270658

[8] Troein P , Newton M , Stoddart K , et al . The impact of Biosimilar competition in Europe. IQVIA; 2022.

[9] Bergstra SA , Branco JC , Vega-Morales D , et al . Inequity in access to bDMARD care and how it influences disease outcomes across countries worldwide: results from the METEOR-Registry. Ann Rheum Dis 2018;77:1413–20. 10.1136/annrheumdis-2018-213289 29980576

[10] Putrik P , Ramiro S , Kvien TK , et al . Inequities in access to biologic and synthetic Dmards across 46 European countries. Ann Rheum Dis 2014;73:198–206. 10.1136/annrheumdis-2012-202603 23467636

[11] Nikiphorou E , van der Heijde D , Norton S , et al . Inequity in biological DMARD prescription for Spondyloarthritis across the globe: results from the ASAS-COMOSPA study. Ann Rheum Dis 2018;77:405–11. 10.1136/annrheumdis-2017-212457 29222349

[12] Lucasson F , Kiltz U , Kalyoncu U , et al . Disparities in healthcare in Psoriatic arthritis: an analysis of 439 patients from 13 countries. RMD Open 2022;8:e002031. 10.1136/rmdopen-2021-002031 35523519 PMC9083399

[13] Putrik P , Ramiro S , Moltó A , et al . Individual-level and country-level socioeconomic determinants of disease outcomes in SpA: multinational, cross-sectional study (ASAS-COMOSPA). Ann Rheum Dis 2019;78:486–93. 10.1136/annrheumdis-2018-214259 30674477

[14] Duggan B , Smith A , Barry M . Uptake of Biosimilars for TNF-Α inhibitors Adalimumab and Etanercept following the best-value biological medicine initiative in Ireland. Int J Clin Pharm 2021;43:1251–6. 10.1007/s11096-021-01243-0 33560486

[15] Aladul MI , Fitzpatrick RW , Chapman SR . The effect of new Biosimilars in rheumatology and gastroenterology specialities on UK Healthcare budgets: results of a budget impact analysis. Res Social Adm Pharm 2019;15:310–7. 10.1016/j.sapharm.2018.05.009 29807834

[16] Goll GL , Kvien TK . An opportunity missed: biosimilars in the United States. Arthritis Rheumatol 2020;72:1046–8. 10.1002/art.41280 32270925

[17] Sullivan SD , Mauskopf JA , Augustovski F , et al . Budget impact analysis—principles of good practice: report of the ISPOR 2012 budget impact analysis good practice II task force. Value in Health 2014;17:5–14. 10.1016/j.jval.2013.08.2291 24438712

[18] WHO . Introduction to DDD indicators. Available: https://www.who.int/tools/atc-ddd-toolkit/indicators [Accessed 20 Aug 2022].

[19] WHO . ATC/DDD index. 2022. Available: https://www.whocc.no/atc_ddd_index [Accessed 20 Aug 2022].

[20] National Health Fund . Resolutions of the national health fund Council. Available: https://www.nfz.gov.pl/zarzadzenia-prezesa/uchwaly-rady-nfz [Accessed 20 Aug 2022].

[21] National Health Fund . Announcements of the Department of drug management of the national health fund. Available: https://www.nfz.gov.pl/aktualnosci/aktualnosci-centrali [Accessed 20 Aug 2022].

[22] National health fund Statistics portal. Available: https://statystyki.nfz.gov.pl [Accessed 20 Aug 2022].

[23] Zangi HA , Ndosi M , Adams J , et al . EULAR recommendations for patient education for people with inflammatory arthritis. Ann Rheum Dis 2015;74:954–62. 10.1136/annrheumdis-2014-206807 25735643

[24] Batko B , Rolska-Wójcik P , Władysiuk M . Indirect costs of rheumatoid arthritis depending on type of treatment-a systematic literature review. Int J Environ Res Public Health 2019;16:2966. 10.3390/ijerph16162966 31426543 PMC6721219

[25] Simoens S , Jacobs I , Popovian R , et al . Assessing the value of biosimilars: a review of the role of budget impact analysis. Pharmacoeconomics 2017;35:1047–62. 10.1007/s40273-017-0529-x 28660473 PMC5606961

[26] Byun HG , Jang M , Yoo HK , et al . Budget impact analysis of the introduction of subcutaneous Infliximab (CT-P13 SC) for the treatment of rheumatoid arthritis in the United Kingdom. Appl Health Econ Health Policy 2021;19:735–45. 10.1007/s40258-021-00673-1 34383287

[27] Kim Y , Kwon H-Y , Godman B , et al . Uptake of Biosimilar Infliximab in the UK, France, Japan, and Korea: budget savings or market expansion across countries. Front Pharmacol 2020;11:970. 10.3389/fphar.2020.00970 32733238 PMC7363801

[28] Brodszky V , Baji P , Balogh O , et al . Budget impact analysis of Biosimilar Infliximab (CT-P13) for the treatment of rheumatoid arthritis in six central and Eastern European countries. Eur J Health Econ 2014;15:65–71. 10.1007/s10198-014-0595-3 PMC4046087 24832837

[29] Aladul MI , Fitzpatrick RW , Chapman SR . Impact of Infliximab and Etanercept Biosimilars on biological disease-modifying Antirheumatic drugs utilisation and NHS budget in the UK. BioDrugs 2017;31:533–44. 10.1007/s40259-017-0252-3 29127626

[30] García-Goñi M , Río-Álvarez I , Carcedo D , et al . Budget impact analysis of Biosimilar products in Spain in the period 2009–2019. Pharmaceuticals (Basel) 2021;14:348. 10.3390/ph14040348 33918795 PMC8069914

[31] Kvien TK , Patel K , Strand V . The cost savings of Biosimilars can help increase patient access and lift the financial burden of health care systems. Semin Arthritis Rheum 2022;52:151939. 10.1016/j.semarthrit.2021.11.009 35027243

[32] Kanters TA , Stevanovic J , Huys I , et al . Adoption of Biosimilar Infliximab for rheumatoid arthritis, Ankylosing Spondylitis, and inflammatory bowel diseases in the EU5: a budget impact analysis using a Delphi panel. Front Pharmacol 2017;8:322. 10.3389/fphar.2017.00322 28620302 PMC5449469

[33] Dörner T , Strand V , Cornes P , et al . The changing landscape of Biosimilars in rheumatology. Ann Rheum Dis 2016;75:974–82. 10.1136/annrheumdis-2016-209166 26964144 PMC4893105

[34] Jensen TB , Kim SC , Jimenez-Solem E , et al . Shift from Adalimumab originator to Biosimilars in Denmark. JAMA Intern Med 2020;180:902–3. 10.1001/jamainternmed.2020.0338 32227137 PMC7105946

[35] Batko B , Stajszczyk M , Świerkot J , et al . Prevalence and clinical characteristics of rheumatoid arthritis in Poland: a nationwide study. Arch Med Sci 2019;15:134–40. 10.5114/aoms.2017.71371 30697263 PMC6348369

[36] Hagège B , Tan E , Gayraud M , et al . Remission and low disease activity in Psoriatic arthritis publications: a systematic literature review with meta-analysis. Rheumatology (Oxford) 2020;59:1818–25. 10.1093/rheumatology/keaa030 32118267

[37] Dean LE , Jones GT , MacDonald AG , et al . Global prevalence of Ankylosing Spondylitis. Rheumatology (Oxford) 2014;53:650–7. 10.1093/rheumatology/ket387 24324212

[38] Scotti L , Franchi M , Marchesoni A , et al . Prevalence and incidence of Psoriatic arthritis: a systematic review and meta-analysis. Semin Arthritis Rheum 2018;48:28–34. 10.1016/j.semarthrit.2018.01.003 29398124

[39] Barcina Lacosta T , Vulto AG , Turcu-Stiolica A , et al . Qualitative analysis of the design and implementation of benefit-sharing programs for Biologics across Europe. BioDrugs 2022;36:217–29. 10.1007/s40259-022-00523-z 35303281 PMC8986662

[40] Batko B , Jeka S , Wiland P , et al . Deep dive into achieving the therapeutic target: results from a prospective, 6-month, observational study nested in routine rheumatoid arthritis care. Pol Arch Intern Med 2022;132:16244. 10.20452/pamw.16244 35420283

[41] Brkic A , Diamantopoulos AP , Haavardsholm EA , et al . Exploring drug cost and disease outcome in rheumatoid arthritis patients treated with biologic and targeted synthetic Dmards in Norway in 2010–2019 – a country with a national tender system for prescription of costly drugs. BMC Health Serv Res 2022;22:48. 10.1186/s12913-021-07425-w 35012522 PMC8743354

