# Supplementary material for: Budget impact analysis and treatment availability with biosimilar TNF inhibitors in rheumatic diseases in Poland: real-world evidence using a nationwide database
Source: Ann Rheum Dis. 2023 Jun 16;82(9):1171–80. doi: 10.1136/ard-2022-223696 (PMC10423465; doi:10.1136/ard-2022-223696)

Supplemental figure 1

Budget impact analysis – retrospective models for assessing the benefits of biosimilars reimbursement.
Both models are based on real drug utilization and the final drug price for hospitals, reflecting the true cost of the public payer.

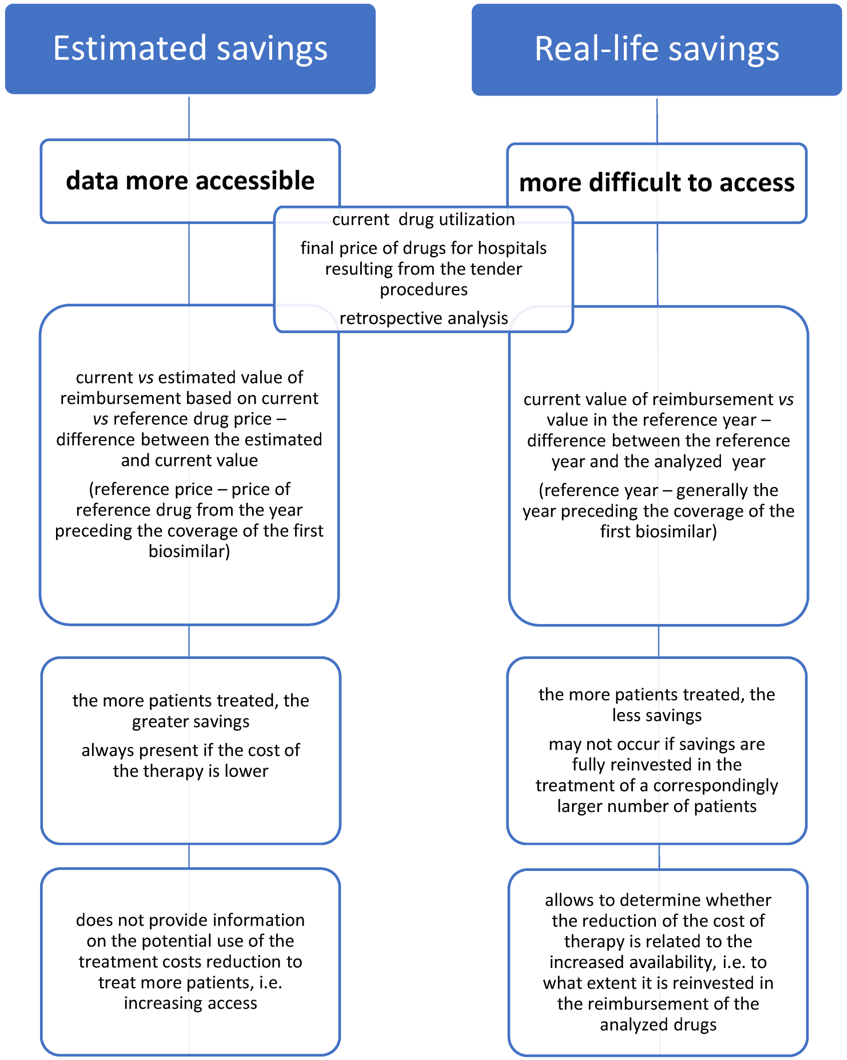

Supplement: Supplementary data [file ard-2022-223696supp001.pdf]
